# The Ultrastructure of Hepatic Stellate Cell–Macrophage Intercellular Crosstalk as a New Morphological Insight into Phenomenon of Fibrogenesis in Pediatric Autoimmune Hepatitis

**DOI:** 10.3390/jcm12031024

**Published:** 2023-01-28

**Authors:** Joanna Maria Łotowska, Maria Elżbieta Sobaniec-Łotowska, Anna Bobrus-Chociej, Piotr Sobaniec

**Affiliations:** 1 Department of Medical Pathomorphology, Faculty of Medicine with the Division of Dentistry and Division of Medical Education in English, Medical University of Bialystok, 15-269 Bialystok, Poland; 2 Department of Pediatrics, Gastroenterology, Hepatology, Nutrition and Allergology, Faculty of Medicine with the Division of Dentistry and Division of Medical Education in English, Medical University of Bialystok, 15-274 Bialystok, Poland; 3 Department of Pediatric Neurology, Faculty of Medicine with the Division of Dentistry and Division of Medical Education in English, Medical University of Bialystok, 15-274 Bialystok, Poland

**Keywords:** pediatric autoimmune hepatitis (AIH), nonparenchymal hepatic cells (NPCs), hepatic stellate cell (HSC) population, transitional hepatic stellate cells (T-HSCs), iHSCs, pHSCs, activated Kupffer cells/macrophages (KCs/MPs), liver fibrogenesis, cellular crosstalk, ultrastructural studies

## Abstract

The aim of the study was the pioneering retrospective ultrastructural evaluation of respective forms of hepatic stellate cells (HSCs) and analysis of their crosstalk with other adjacent nonparenchymal cells (NPCs), especially Kupffer cells/macrophages (KCs/MPs), in pediatric autoimmune hepatitis (AIH). Methods: Ultrastructural assessment of the HSC population and NPCs was performed in transmission electron microscopy (TEM) using pretreatment liver biopsies from 25 children (8 boys and 17 girls) aged 4–17 with clinic-pathologically diagnosed untreated AIH. Results: Submicroscopic evaluation allowed easy identification of numerous HSCs in the form of transitory cells, i.e., T-HSCs, accompanied by signs of fibrosis. T-HSCs included cells with features of activation initiation (iHSCs) and activation perpetuation (pHSCs), indicating high HSC activation plasticity. The pHSCs were markedly elongated and mainly showed a distinct loss of lipid cytoplasmic material, expanded and dilated channels of granular endoplasmic reticulum, and linear bundles of microfilaments beneath the cell membrane. They were surrounded by usually mature collagen fibers. Frequently activated KCs/MPs adhered directly to T-HSCs. Between them, tight intercellular junctions were formed by means of point desmosomes. Conclusions: Our qualitative TEM observations indicate a key role of T-HSCs in liver fibrogenesis in pediatric AIH, with the essential involvement of activated KCs/MPs that directly adhere to them. Tight intercellular junctions, being the ultrastructural exponent of the specific cellular mechanisms of the crosstalk between NPCs, can play a vital role in hepatic collagen fibroplasia. A better understanding of HSC population morphology at the ultrastructural level in AIH seems important not only to improve the disease morphological diagnostics but to also provide new insights into therapeutic interventions for the phenomenon of liver fibrogenesis.

## 1. Introduction

Autoimmune hepatitis (AIH) is an immune-mediated autodestructive inflammatory disease affecting both children and adults, with female predominance, in the absence of a known etiology. Its diagnosis remains difficult and should involve clinical manifestations, laboratory evaluation, histopathology, and exclusion of other more common liver diseases. It is assumed that inflammatory liver histopathology manifested by interface hepatitis and plasma cell infiltration as hallmark lesions, elevated transaminase levels, circulating non-organ-specific autoantibodies, and increased levels of immunoglobulin G is the most characteristic of the disease [1,2,3,4,5,6,7]. AIH is particularly aggressive in children/adolescents, progressing rapidly without early initiation of immunosuppressant therapy [4,6,8,9,10,11].

Many hepatologists emphasize that interface hepatitis in this liver disorder is closely related to liver fibrosis, leading in some cases to cirrhosis, liver failure, and death [9,10,11,12,13,14,15]. Microscopic investigations have been long conducted to elucidate the complex morphological nature of the process of fibrogenesis and fibrosis progression in chronic liver diseases. These investigations are crucial not only for diagnostic purposes but also to search for antifibrotic therapy. Unfortunately, there have been no morphological reports explaining this important phenomenon at the ultrastructural level in the course of AIH.

It is assumed that hepatic fibrosis and end-stage liver cirrhosis, irrespective of the injury factor that induced fibrosis, are dynamic wound-healing processes characterized by the deposition of the extracellular matrix (ECM), activation of cells capable of producing matrix materials, cytokine release [16,17,18,19,20,21,22], and tissue remodeling regulated by matrix metalloproteinases and their inhibition [19,20,23,24,25,26]. Unfortunately, no efficient antifibrotic therapy, excluding liver transplantation, has been available so far.

Many researchers have noted that activated hepatic stellate cells (HSCs)/myofibroblasts, especially the transitional form of HSCs (T-HSCs), constitute the main fibrogenic cell type in the liver [17,18,21,23,27,28,29]. It has been assumed that HSCs, apart from liver sinusoidal endothelial cells (LSECs), hepatic macrophages (Kupffer cell/macrophages—KC/MPs), and a heterogeneous subpopulation of intrahepatic lymphocytes, belong to the population of nonparenchymal hepatic cells (NPCs) [22,29,30,31,32].

HSCs are easy to identify using transmission electron microscopy (TEM) or immunohistochemical (IHC) stainings, especially for smooth muscle actin (alpha-SMA) and desmin and some neuronal markers such as glial fibrillary acidic protein and synaptophysin [23,28,29,33,34], but not with routine histological staining.

Ultrastructural investigations allowed researchers to distinguish three main morphological forms of HSCs—quiescent HSCs (Q-HSCs), transitional HSCs (T-HSCs), and myofibroblastic HSCs (Mf-HSCs)—i.e., fibrogenic myofibroblast-like cells. It is assumed that in the normal liver, HSCs are quiescent and represent 5–8% of the total number of liver cells. They are known for vitamin A (retinoid) storage in their cytoplasmic droplets as vitamin A-storing cells [23,26,27,29,32,34,35].

It is now widely believed that HSCs, which undergo transformation to metabolically active T-HSCs and myofibroblast-type cells, i.e., Mf-HSCs, are mainly responsible for the synthesis of protein components of the extracellular matrix (ECM) and play a crucial role in the initiation and progression of liver fibrosis [17,18,19,21,27,29,35]. They are also a potential target for the treatment of liver fibrosis [27,36,37,38,39,40,41,42]. Thus, T-HSCs constitute a major source of fibril-forming ECM in the liver. 

Paracrine signals from resident cells such as hepatocytes, liver sinusoidal endothelial cells, Kupffer cell/macrophages, natural killer/natural killer T cells, biliary epithelial cells, hepatic progenitor cells (HPCs), and platelets can directly or indirectly regulate HSC differentiation and activation [18,21,23,27,31,35,43,44]. These cells can precisely ”talk” to each other as stated by Cai et al. (2020) [27] and also by other authors in their latest works on the role of activated HSCs in the process of liver fibrosis [17,21,22,27,29,35]. We would like to emphasize here the significance of an interesting comprehensive and still up-to-date work of a Polish researcher Kmieć who, 20 years ago, reported on the cooperation of hepatocytes and nonparenchymal hepatic cells in health and disease. That study, being the foundation for the concept of “cellular cross-talk”, inspired our current ultrastructural investigations of fibrogenesis in pediatric AIH [31].

It should be added that most hepatological studies conducted so far devoted to the process of liver fibrogenesis, which occurs in various chronic liver diseases, are mainly supported by repeated schematic drawings without presenting the electron microscopic documentation of this phenomenon.

Up to now, literature data on the sequence of morphological events observed at the ultrastructural level in the pathogenesis of liver fibrosis have been limited to adult patients and experimental studies. Similar reports on pediatric patients are lacking.

Taking that into consideration, the aim of the current study was to provide, for the first time in the hepatological literature, a descriptive qualitative morphological research characterizing the ultrastructural profile of the respective forms of hepatic stellate cells and adhering hepatic macrophages in untreated children with AIH, in relation to the process of fibrogenesis, based on retrospective biopsy material. Also interesting is the submicroscopic analysis of the intercellular crosstalk between HSCs and adjacent nonparenchymal hepatic cells, especially KCs/MPs, in the same pediatric patients with AIH. The elucidation of the ultrastructure characteristics of this intercellular communication of cell–cell interaction in HSC activation may be crucial for research on novel potential antifibrotic therapies in AIH.

The current study is a continuation of our many years of morphological and clinical research, especially ultrastructural studies in chronic liver diseases in children, such as chronic hepatitis B (chB) [28,29,45,46,47], nonalcoholic steatohepatitis (NASH) [48,49], pediatric AIH [3,50,51], and also in the formation of biliary fibrosis in an animal experimental model [44]. It is noteworthy submicroscopic and immunohistochemical observations with a focus on the role of the population of NPCs and HPCs in the morphogenesis of pediatric AIH [3,50,51].

## 2. Material and Methods

### 2.1. Study Patients’ Profile

Retrospective ultrastructural investigations were conducted using tissue material embedded in epon blocks, including pretreatment needle liver biopsy specimens obtained from 25 untreated children (8 boys and 17 girls) aged 4–17 hospitalized in the Department of Pediatrics, Gastroenterology, Hepatology, Nutrition and Allergology, Medical University of Bialystok, with clinic-pathologically diagnosed AIH.

The laboratory tests revealed markedly increased serum levels of aspartate and alanine aminotransferase in all study patients. Immunological and serological disturbances in the blood serum were manifested by substantially elevated IgG levels and the presence of autoantibodies—antinuclear antibodies (ANAs) and/or smooth muscle antibodies (SMAs). Differential diagnostics excluded, among others, infectious liver diseases (HBV, HCV, CMV, and Toxoplasma gondii), some metabolic disorders (Wilson’s disease, cystic fibrosis, and alpha-1 antitrypsin deficiency), and celiac disease. The clinical data, including immunological and serological disturbances in blood serum and differential diagnostics of patients, were reported in our earlier papers [3,50].

The collected material was subjected to pathomorphological investigations, i.e., histological, histochemical, immunohistochemical (for alpha-SMA and desmin, CK7 and CK19), and ultrastructural analyses using TEM in the Department of Medical Pathomorphology, Medical University of Bialystok.

It should be added that as regards the identification of two subpopulations of liver macrophages (KCs/MPs) carried out in the present study at the level of electron microscopy, it was easy to distinguish KCs, i.e., liver resident macrophages, from the others, i.e., liver-recruited macrophages. KCs showed their characteristic location within the lumen of the liver sinusoides and also the submicroscopic image. In contrast, for the remaining liver macrophages, similar to other authors dealing with the ultrastructure of this cell population and to our previous research on KCs/MPs in AIH [3], we treated them as liver-recruited macrophages.

Hepatic necroinflammatory injuries and fibrosis had been previously assessed histologically using routine Mayer’s hematoxylin and eosin (H&E) stain. Liver fibrosis staining for connective tissue stroma components was determined by a panel of histochemical stains (for collagen fibers, by Sirius red, Masson’ trichrome blue, the Azan method, according to Masson–Goldner, and for reticulin fibers, according to Gomori) and assessed by a single hepatopathologist blinded to patient clinical data. The study showed typical histological features of AIH, i.e., interface and lobular hepatitis, moderate/severe in nature with portal infiltration of lymphocytes and plasma cells, severe necroinflammatory reaction, and rosette formation of hepatocytes. The alterations were frequently accompanied by portal, periportal, and bridging fibrosis [3,50,51]. The stage of fibrosis (staging—S; range: 0–4) and inflammation grade (grading—G; range: 0–4) in liver biopsies were retrospectively scored using the semiquantitative scoring system according to Batts and Ludwig [52] and, in some cases, complemented by the scoring system proposed by Ishak [53]. In the group of 25 children, we identified 5 patients with advanced liver fibrosis (corresponding to S-3) but not with liver cirrhosis and 7 with mild (S-1) and 13 with moderate (S-2) liver fibrosis.

It should be notice that, for obvious bioethical and procedural reasons, no control groups consisting of healthy children subjected to liver biopsy could be formed as liver biopsy is an invasive procedure and cannot be performed in control, healthy children. This is clearly stated by the ESPGHAN Hepatology Committee in the guidelines of 2015 for pediatric liver biopsy [4]. Thus, we compared the ultrastructure of the respective morphological forms of HSCs in pediatric AIH to the same cell population in other chronic liver diseases in children investigated at our center, i.e., chronic hepatitis B [29,45,47] and nonalcoholic steatohepatitis (NASH) [48,49].

Informed consent was obtained from the parents of each patient included in the study. The current research was approved by the Ethical Committee, Medical University of Bialystok (R-I-002/410/2016).

### 2.2. Liver Tissue Processing for Transmission Electron Microscopy

For TEM, fresh small tissue blocks (1 mm^3^ volume) obtained from liver biopsy material were primarily fixed in Karnovsky’s fixative (2% paraformaldehyde and 2.5% glutaraldehyde in 0.1 M cacodylate buffer, pH 7.4) for 12 h. Next, they were postfixed in 2% osmium tetroxide (OsO4) in 0.1 M cacodylate buffer (pH 7.4) for 1 h. Then, the material was dehydrated through a graded series of ethanols and propylene oxide, embedded in Epon 812 or Gycid ether 100 for electron microscopy, and sectioned on a Reichert ultramicrotome (Reichert Ultracut S). The obtained semithin sections were stained with 1% methylene blue in 1% sodium borate. The ultrathin sections (approximately 70 nm) were subsequently cut with the same Reichert ultramicrotome, mounted on copper grids, contrasted with uranyl acetate and lead citrate, examined under an Opton EM 900 electron microscope (Oberkochen, Germany), and photographed with a TRS camera (CCD—camera for TEM, 2K inside). The same procedure had been used in our earlier ultrastructural investigations of the liver in children [3,29,50,51].

The ultrastructural profile of the HSC population and the surrounding cells in relation to the process of fibrogenesis was determined by a microscopist who was blinded to the clinical information.

## 3. Results 

**Figure 1:**
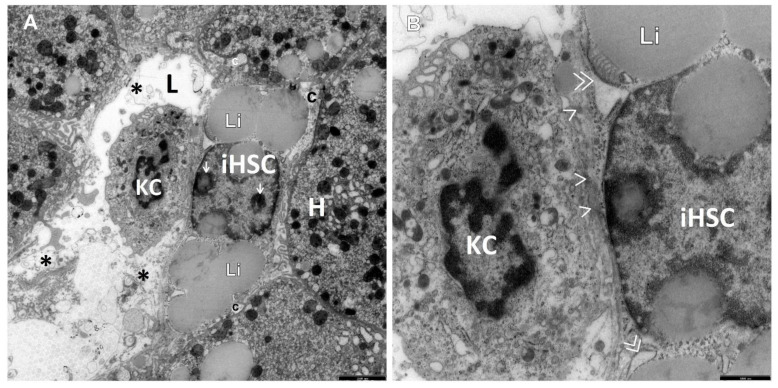
(**A**,**B**) A picture of a slightly elongated Q-HSC being transformed into T-HSC, which may correspond to the phase of activation initiation, i.e., iHSC, and an activated Kupffer cell (KC) that adheres tightly to it from the side of the sinusoidal vascular lumen in liver biopsy obtained from a child with AIH (S-1). Between these cells, there is a direct cell–cell contact by means of desmosomes (>), well visible at a larger magnification (**B**). The cell nucleus contains two activated nucleoli (->); the cell is rich in lipid material; and the cytoplasm shows slightly dilated granular endoplasmic reticulum (ger) channels (>>). A small number of mature collagen fibers (c) adhere focally to the iHSC. The vascular pole of hepatocyte (H) sends numerous microvilli towards the HSC. In the vicinity of KC, the sinusoid vascular lumen shows fragments of damaged liver sinusoidal endothelial cells (*). L—the lumen of liver sinusoid. Scale bar 2.5 μm, original magnification ×4400 (**A**); 1 μm, original magnification ×12,000 (**B**).

**Figure 2:**
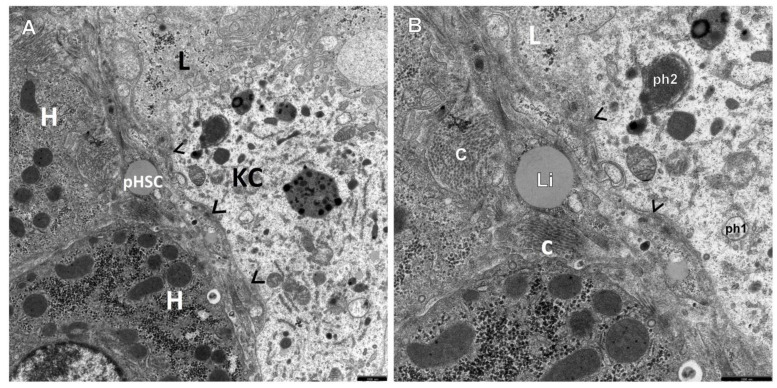
(**A**,**B**) A picture of a markedly elongated perisinusoidal pHSC adhering to the smoothed vascular pole of the hepatocyte (H). The T-HSC has very thin cytoplasmic processes and contains a single lipid droplet (Li) and dilated ger components. An activated and markedly swollen Kupffer cell (KC), containing primary (ph1) and secondary (ph2) phagosomes, adheres directly to the pHSC from the side of the sinusoidal vascular lumen (L). Between the smoothed vascular pole of the hepatocytes directed towards the perisinusoidal space and the pHSC, there are tiny bundles of mature collagen fibers (c). Larger magnification demonstrates tight intercellular junctions between pHSC and KC (>). Scale bar: 1 μm, original magnifications: ×7000 (**A**), ×12,000 (**B**).

**Figure 3:**
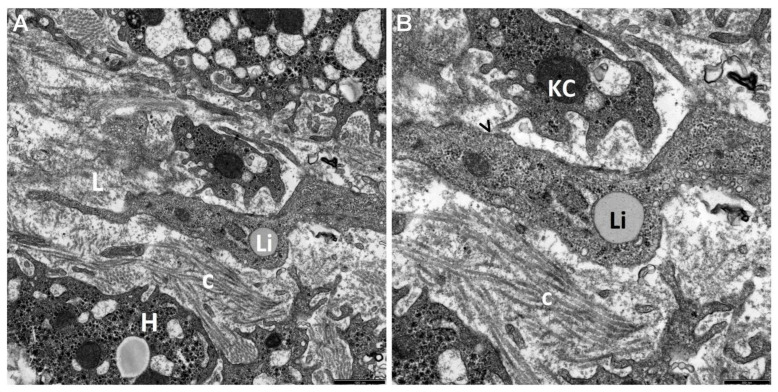
(**A**,**B**) An electronogram demonstrating a fragment of an elongated perisinusoidal stellate pHSC containing a single lipid droplet (Li), weakly dilated ger channels, and a relatively well-preserved mitochondrion. A fine cytoplasmic process (>) of a Kupffer cell (KC) adheres directly to the upper pole of the pHSC; the lower pole is enclosed by a bundle of mature collagen fibers—c (visible is longitudinal section through these fibers). The vacular pole of the hepatocyte (H) is smoothed in places; L—sinusoidal vascular lumen. Scale bar 1 μm, original magnification ×7000 (**A**); scale bar 0.5 μm, original magnification ×12,000 (**B**).

**Figure 4:**
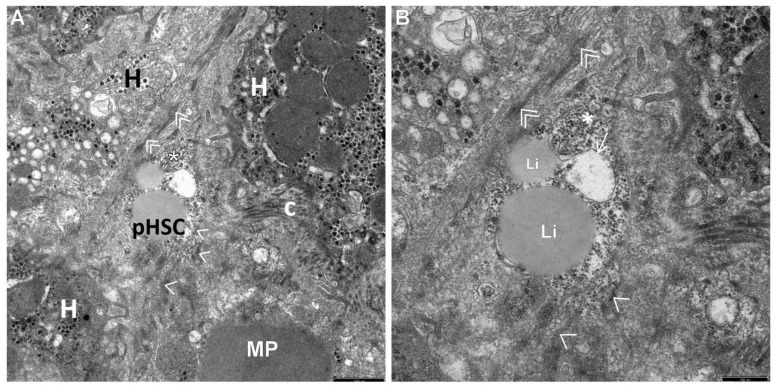
(**A**,**B**) An ultrastructural picture of a T-HSC, with features of pHSC, which lost a part of lipid material (Li) and is surrounded by hepatocytes (H) and an activated macrophage (MP). A tiny bundle of collagen fibers (c) is seen to adhere to the pHSC. The pHSC cytoplasm shows section through a markedly cistern-like dilated ger channel (->), almost completely deprived of ribosomes and with fine microfibrillar material; around this ger channel, there are areas of the cytoplasm rich in free ribosomes (*); also visible are submembranous linearly accumulating bundles of microfilaments (>>) (i.e., submembranous dense areas). Between the pHSC and MP, there are tight intercellular junctions (>). Scale bar 1 μm, original magnification ×7000 (**A**); scale bar 0.5 μm, original magnification ×12,000 (**B**).

**Figure 5:**
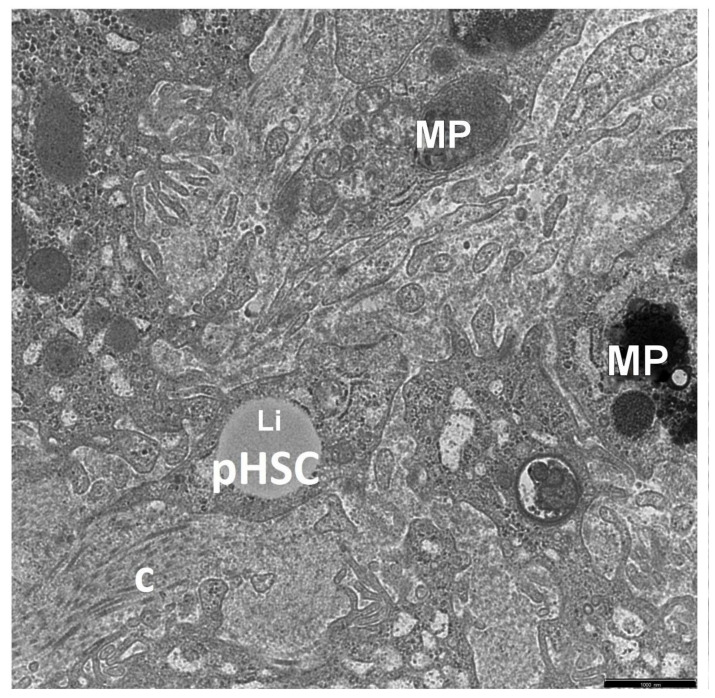
An electronogram demonstrating a pHSC, centrally located in the space between hepatocytes, elongated and containing a relatively large single lipid droplet (Li). This cell is surrounded by thick bundles of collagen fibers (c). In very close vicinity, there are fragments of cells resembling activated Kupffer cells/macrophages (MPs) outside the sinusoids. Scale bar 1 μm, original magnification ×12,000.

**Figure 6:**
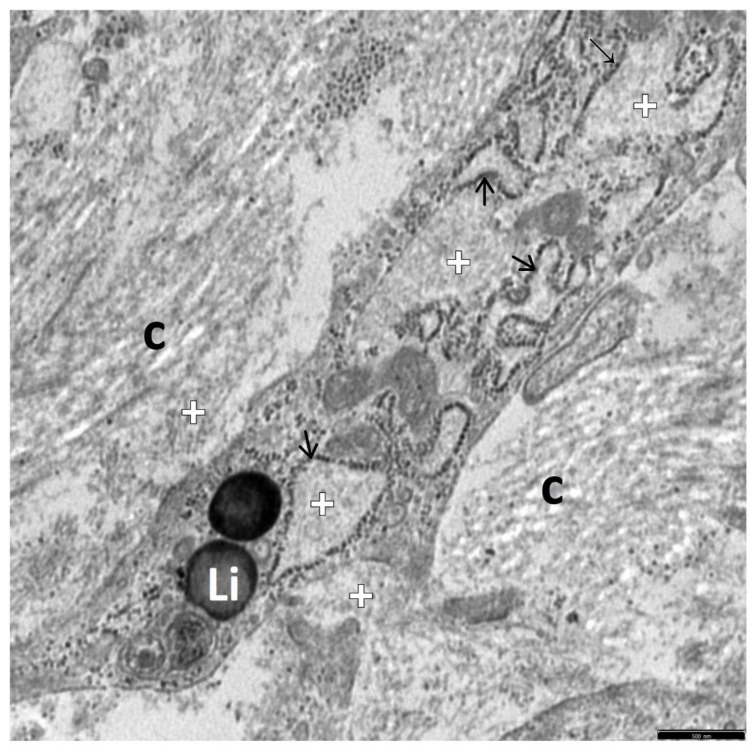
An electronogram of a fragment of a pHSC situated between thick bundles of mature collagen fibers (c). Its cytoplasm shows markedly dilated ger channels (->); visible areas of accumulating microfibrillar, flocculent material within substantially dilated ger channels, loosely laying outside them, and in some places, directly adhering to pHSC (+), which may correspond to deposits of immature collagen components; Li—fine single lipid droplets. Scale bar 0.5 μm, original magnification ×20,000.

The retrospective ultrastructural analysis of the liver affected by AIH from children with coexisting mild fibrosis (S-1) showed the transformation of resting forms of stellate cells, i.e., Q-HSCs, normally found in a healthy liver, into a transitory form of hepatic stellate cells (T-HSCs) in the phase of activation initiation (i-HSCs) (Figure 1A,B). Interestingly, T-HSCs being in the phase of activation perpetuation, i.e., pHSCs were also found, although more seldom, in AIH coexisting with mild fibrosis (Figure 2A,B). However, the analogous electron microscopic study using pretreatment liver biopsies obtained from children with AIH and accompanying advanced fibrosis (S-2, S-3) showed almost a complete lack of resting forms of stellate cells and their replacement with transitory hepatic stellate cells presenting features of pHSCs (Figure 3A,B, Figure 4A,B, Figure 5 and Figure 6).

In Figure 2, Figure 3, Figure 4, Figure 5 and Figure 6, electron micrographs show perisinusoidally located pHSCs, i.e., T-HSCs in the phase of activation perpetuation, impacted between the smoothed vascular pole of the hepatocyte (H) and liver sinusoid (Figure 2A,B and Figure 3A,B) and T-HSCs lying in the intercellular septa of hepatocytes (Figure 4A,B, Figure 5 and Figure 6) with the accompanying process of fibrosis in liver biopsy obtained from children with AIH. S: 1 (Figure 2A,B); 2 (Figure 3A,B and Figure 4A,B); 3 (Figure 5 and Figure 6).

The cell nucleus of iHSCs contained a distinct nucleolus/nucleoli, while the cytoplasm showed an abundance of lipid material and slightly dilated channels of granular endoplasmic reticulum (ger), which is well demonstrated in Figure 1A,B.

It should be noted that TEM examination allowed easy identification of numerous second-type T-HSCs, i.e., in the phase of activation perpetuation, which clearly predominated in the whole population of the hepatic stellate cells studied.

The subpopulation of pHSCs showed either perisinusoidal location (Figure 2A,B and Figure 3A,B) or was observed outside the space of Disse, in the spaces between hepatocytes (Figure 4A,B, Figure 5 and Figure 6). These cells were markedly elongated (Figure 2A,B, Figure 3A,B and Figure 6). Many times, their substantially elongated and thinned cytoplasmic processes reached neighboring hepatocytes (Figure 2A,B). Interestingly, pHSCs showed a substantial loss of lipid cytoplasmic material (Figure 2A,B, Figure 3A,B and Figure 6) and frequently contained only a single drop or very tiny lipid droplet, with a volume smaller than 10% of the cytoplasmic volume (Figure 3A,B and Figure 6). The cytoplasm of such cells contained expanded and markedly dilated channels of ger with the formation of cistern-like structures filled up with delicate flocculent material, segmentally deprived of ribosomes (Figure 4A,B and Figure 6). Greater degranulation of ger channels resulted in the formation of cytoplasm areas filled with free ribosomes (Figure 4A,B). Sometimes, dilated canals of the Golgi apparatus were observed. Cytoskeleton components were observed as linearly arranged bundles of microfilaments, usually around 5 nm in diameter, beneath the cell membrane of pHSCs (Figure 4A,B).

Many times, pHSCs were surrounded by abundant quantities of thick bundles of mostly mature collagen fibers that frequently adhered directly to them (Figure 3A,B, Figure 5 and Figure 6). However, apart from mature collagen fibers, some images also showed a microfibrillar, flocculent, and condensed material in the cytoplasm of pHSCs (within the markedly dilated channels of ger and loosely laying outside them) and in very close vicinity of these cells, which can be referred to as a morphological precursor of collagen fibers (Figure 6).

Interestingly, activated KCs/MPs directly adhered to T-HSCs (both to iHSCs and to pHSCs). These cells were markedly enlarged and contained numerous primary and secondary phagosomes that varied in size and were filled up with the phagocytized extracellular material (Figure 1A,B, Figure 2A,B, Figure 4A,B and Figure 5).

The adhering T-HSCs and activated KCs/MPs formed direct cell–cell contacts, which was ultrastructurally demonstrated by the presence of tight intercellular junctions (point desmosomes) (Figure 1A,B, Figure 2A,B and Figure 4A,B).

It should be added that the comparison of the current findings presenting the ultrastructural profile of the respective morphologic forms of HSCs in relation to the process of fibrogenesis in pediatric AIH with the same cell population in other chronic liver diseases in children assessed in our earlier studies in TEM revealed that it was not specific to this disease, since it considerably resembled the ultrastructure of these cells in the course of chronic hepatitis B [29,45,47] and NASH [48,49] in pediatric patients hospitalized at our center.

## 4. Discussion

The currently conducted, for the first time in hepatology, electron microscopic study of a population of stellate cells of the liver in pediatric AIH coexisting with fibrogenesis and fibrosis progression revealed almost complete replacement of Q-HSCs, i.e., vitamin A-storing cells, by T-HSCs, which were in two activation phases: initiation and perpetuation, indicating high plastic properties of this cell population.

The pHSCs, which were the most commonly observed in our study, were markedly elongated and showed substantial loss of lipid cytoplasmic material and the presence of markedly delated channels of ger, segmentally deprived of ribosomes, within the cytoplasm. Beneath the cell membrane, there were linearly arranged bundles of microfilaments. These cells were frequently surrounded by abundant, mostly mature collagen fibers forming bundles. Many times, activated KCs/MPs directly adhered to or were in close vicinity of T-HSCs. Between the adhering T-HSCs and liver macrophages, there were direct cell–cell contacts, i.e., intercellular junctions constituting the morphological sign of cell–cell interactions.

Interestingly, the submicroscopic picture of the HSC population and fibrogenesis in pediatric AIH did not show features of morphological specificity to this pathology since its ultrastructure considerably resembled the ones observed in chB [29,45,47] and NASH [48,49] in children. 

Literature data emphasize that, in the process of liver fibrogenesis, Q-HSCs become activated, gradually reducing the vitamin A storage capacity, proliferating, developing a contractile function, secreting excessive ECM proteins, and also releasing a series of proinflammatory (e.g., interleukin 6 [IL6] and profibrogenic factors, e.g., transforming growth factor beta (TGF-β)) [16,19,31,35,54,55].

Generally, HSC activation occurs in two phases: initiation and perpetuation. In the initiation period, Q-HSCs receive molecular signals from adjacent cells, which secrete profibrogenic cytokines and growth factors, and then they demonstrate profibrogenic transcriptional and secretory properties. If activated HSCs proliferate and constantly secrete ECM proteins, leading to the accumulation of ECM and the formation of scar tissue, they are in the phase of perpetuation. The perpetuation of HSCs involves a number of cell–cell interactions and wound-healing responses [16,17,18,21,27,29,32]. If fibrosis begins to regress, the activated HSCs may develop a potential third phase, i.e., resolution, where they may either undergo apoptosis or revert to the senescent/quiescent phenotype [17,18,23,31,32,37].

It should be emphasized that HSC activation is a dynamic process that mainly depends on the interaction with other adjacent cells, including hepatocytes, hepatic macrophages, liver sinusoidal endothelial cells, natural killer/natural killer T (NK/NKT) cells, biliary epithelial cells, heptic progenitor cells (HPCs), and platelets, which can precisely “talk” to each other [17,21,22,27,29,31,45].

The exact ultrastructural picture of activated KCs/MPs, LSECs, and HPCs that may help in the diagnosis of pediatric AIH was presented in our earlier manuscripts devoted to this pathology [3,50,51].

Hepatic macrophages, in a simplified way and taking into account their ontogenes, can be divided into liver resident macrophages, termed Kupffer cells (KCs), and blood- or bone-marrow-derived macrophages recruited to the liver, called monocyte-derived macrophages (MPs). KCs are localized within the lumen of the liver sinusoids, accounting for about 30% of sinusoidal cells [30].

It is currently assumed that Kupffer cells, which adhere to the sinusoidal endothelial layer, can be activated by circulating diverse stimuli of blood and secreting various proinflammatory cytokines and chemokines, including TGF-β1, TNF-α, MCP-1, CCL3, and CCL5, and other soluble mediators, inducing a physiological response to other liver cells [21,27,31,42,56,57,58,59]. There is clear evidence from in vitro and in vivo studies that KCs can activate HSCs into the transitional form of HSCs, i.e., T-HSCs, the major collagen-producing cell type in liver fibrogenesis. KCs activate hepatic stellate cells via paracrine mechanisms, likely involving the potent profibrotic and mitogenic cytokines TGF-β and PDGF [18,56].

It is worth noting that although it is well established that hepatic macrophages, including Kupffer cells and recruited macrophages, play a crucial role in the process of fibrogenesis and in the progression of liver fibrosis, the underlying mechanisms remain largely elusive and poorly understood. Therefore, the interesting research conducted by Pradere and Colleagues on the mechanisms of macrophage-HSC crosstalk deserves special attention here, which clearly showed that hepatic macrophages bilaterally regulate the activation of HSCs [60]. The authors demonstrated, for the first time in hepatology, that hepatic macrophages enhance liver fibrosis by promoting the survival of activated HSCs in a nuclear factor kappa B (NF-ĸB)-dependent manner. Moreover, they showed that macrophage-induced activation of NF-ĸB in HSCs in vitro and in vivo was mediated by interleukin (IL)-1 and tumor necrosis factor. They emphasized that IL-1 and TNF did not promote HSC activation but promoted the survival of activated HSCs in vitro as well as in vivo and thereby increased liver fibrosis. However, the neutralization of these proinflammatory cytokines in coculture experiments and genetic ablation of their receptors in mouse models led to increased apoptosis of HSCs and decreased fibrosis [60].

It is also worth adding that inhibition of NF-ĸB (via inhibition of the inhibitor of ĸB kinase/nuclear factor-ĸB pathway) with sulfasalazine induced apoptosis of activated rat and human HSCs, exerting a potential antifibrotic effect [61].

Like other authors, we believe that it is necessary to identify the key triggers between KC activity and HSC activation in future research, which may contribute to more effective interventions for KCs in liver fibrosis [18,21,22,27,31,58,62,63]. It is assumed that some important proteins can regulate the cooperation between KCs and HSCs [21,31,57,58,59,62,63], which may benefit the therapy of liver fibrosis. For instance, Oncostatin M (OSM), a member of the IL-6 family cytokines, triggers collagen production in HSCs depending on the cellular source of fibrogenic cytokines from hepatic macrophages [62]. When macrophage regulation is blocked on OSM, collagen secretion of HSCs is inhibited, thus leading to reduced fibrosis.

Although the results of some experimental studies seem to be optimistic, showing that liver fibrosis is reversible and even that cirrhosis may regress in some cases, there is no effective antifibrotic therapy [27,36,37,38,40,41,42,64,65]. Responsible for this situation is probably a relatively small number of morphological studies, especially submicroscopic investigations, in this field. It is, therefore, important to identify the vital pathological mechanisms underlying liver fibrogenesis, which could hopefully develop more efficient antifibrotic therapies to improve the quality of life of patients with liver fibrosis, including AIH. Deciphering the morphogenesis of the specific cellular mechanisms, taking into account the ultrastructure of cell–cell interactions in HSC activation, may provide new insights into therapeutic strategies for hepatic fibrosis. We still do not know what plays the leading role in the regulation of HSC activation in the process of liver fibrosis.

As the intercellular crosstalk in liver fibrogenesis in pediatric AIH is poorly understood, improved knowledge of the submicroscopic picture of cellular communication, i.e., cell–cell interactions between HSCs and the surrounding cells, especially NPCs, could more efficiently promote fibrosis resolution and liver regeneration in the future. We hope that our current ultrastructural qualitative study, being the first to investigate the process of fibrogenesis in children with AIH, constitutes interesting comparative material for similar observations in children with this pathology. Further morphological research on the phenomenon of liver fibrogenesis in pediatric AIH will be also extended to quantitative analyses at our center.

## 5. Conclusions

The current ultrastructural qualitative assessment of hepatic stellate cells in children with clinic-pathologically diagnosed AIH allowed the identification of numerous transitional HSCs in the place of normal quiescent HSCs, with accompanying marked features of fibrogenesis. T-HSCs showed features of initiation and perpetuation of activation (i.e., iHSCs and, the most predominant, pHSCs), which may indicate high properties of fibroblastic plasticity of this cell population. Many times, the activated KCs/MPs directly adhered to T-HSCs by means of the so-called point desmosomes. Tight intercellular junctions, being the ultrastructural component of the specific cellular mechanisms of crosstalk between NPCs, can play a vital role in the process of liver fibrogenesis in AIH.

In our opinion, further clarification of these cellular mechanisms in the population of hepatic stellate cells in children with AIH at the level of TEM, including cell–cell interaction in HSC activation, seems crucial not only for thorough microscopic diagnostics of the disease that is also extended with morphogenesis and fibrosis progression but also for providing new insights into therapeutic interventions for the phenomenon of liver fibrosis.

## Data Availability

Not applicable.

## References

[1] Mieli-Vergani G., Vergani D., Czaja A.J., Manns M.P., Krawitt E.L., Vierling J.M., Lohse A.W., Montano-Loza A.J. (2018). Autoimmunehepatitis. Nat. Rev. Dis. Prim..

[2] Cha H.J., Hwang J., Lee L.E., Park Y., Song J.J. (2021). The significance of cytoplasmic antinuclear antibody patterns in autoimmune liver disease. PLoS ONE.

[3] Lotowska J.M., Sobaniec-Lotowska M.E., Daniluk U., Lebensztejn D.M. (2017). Glassy droplet inclusions within the cytoplasm of Kupffer cells: A novel ultrastructural feature for the diagnosis of pediatric autoimmune hepatitis. Dig. Liver Dis..

[4] Dezsőfi A., Baumann U., Dhawan A., Durmaz O., Fischler B., Hadzic N., Hierro L., Lacaille F., McLin V.A., Nobili V. (2015). Liver 533 biopsy in children: Position paper of the ESPGHAN Hepatology Committee. J. Pediatr. Gastroenterol. Nutr..

[5] Tanaka A. (2020). Autoimmune Hepatitis: 2019 Update. Gut Liver.

[6] Arcos-Machancoses J.V., Busoms C.M., Tatis E.J., Bovo M.V., Bernabeu J.Q., Goñi J.J., Martínez V.C., de Carpi J.M. (2019). Development and validation of a new simplified diagnostic scoring system for pediatric autoimmune hepatitis. Multicenter Study. Dig. Liver Dis..

[7] Fujiwara K., Fukuda Y., Seza K., Saito M., Yasui S., Nakano M., Yokosuka O., Kato N. (2018). Long-term observation of acute-onset autoimmune hepatitis presenting clinically and radiologically as acute hepatitis. Hepatol. Int..

[8] Sokollik C., McLin V.A., Vergani D., Beretta-Piccoli B.T., Mieli-Vergani G. (2018). Juvenile autoimmune hepatitis: A comprehensive review. J. Autoimmun..

[9] Puustinen L., Barner-Rasmussen N., Pukkala E., Färkkilä M. (2019). Incidence, prevalence, and causes of death of patients with autoimmune hepatitis: A nationwide register-based cohort study in Finland. Dig. Liver Dis..

[10] Nares-Cisneros J., Jaramillo-Rodríguez Y. (2014). Autoimmune hepatitis in children: Progression of 20 cases in northern Mexico. Rev. Gastroenterol. Mex..

[11] Floreani A., Liberal R., Vergani D., Mieli-Vergani G. (2013). Autoimmune hepatitis: Contrasts and comparisons in children and adults—A comprehensive review. J. Autoimmun..

[12] Soares J.C., Borgonovo A., Maggi D.C., Pasinato A.P., Ramos F.G., Dantas-Corrêa E.B., Schiavon L.L., Narciso-Schiavon J.L. (2016). Liver dysfunction and fibrosis as predictors of biochemical response to autoimmune hepatitis treatment. Minerva Gastroenterol. Dietol..

[13] Fujiwara K., Yasui S., Yokosuka O. (2013). Autoimmune acute liver failure: An emerging etiology for intractable acute liver failure. Hepatol. Int..

[14] Lohse A.W., Sebode M., Bhathal P.S., Clouston A.D., Dienes H.P., Jain D., Gouw A.S.H., Guindi M., Kakar S., Kleiner D.E. (2022). Consensus recommendations for histological criteria of autoimmune hepatitis from the International AIH Pathology Group: Results of a workshop on AIH histology hosted by the European Reference Network on Hepatological Diseases and the European Society of Pathology. Liver Int..

[15] Griessmair L., Pirringer L., Mountford S., Sendelhofert A., Makeschin M.C., Koletzko S., Mayr D., Bufler P. (2022). Expression of IL-37 Correlates with Immune Cell Infiltrate and Fibrosis in Pediatric Autoimmune Liver Diseases. J. Pediatr. Gastroenterol. Nutr..

[16] Zhou W.C., Zhang Q.B., Qiao L. (2014). Pathogenesis of liver cirrhosis. World J. Gastroenterol..

[17] Zhang C.Y., Yuan W.G., He P., Lei J.H., Wang C.X. (2016). Liver fibrosis and hepatic stellate cells: Etiology, pathological hallmarks and therapeutic targets. World J. Gastroenterol..

[18] Tsuchida T., Friedman S.L. (2017). Mechanisms of hepatic stellate cell activation. Nat. Rev. Gastroenterol. Hepatol..

[19] Pinzani M. (2015). Pathophysiology of liver fibrosis. Dig. Dis..

[20] Helal T.E.S.A., Ehsan N.A., Radwan N.A., Abdelsameea E. (2018). Relationship between hepatic progenitor cells and stellate cells in chronic hepatitis C genotype 4. APMIS.

[21] Yang F., Li H., Li Y., Hao Y., Wang Ch., Jia P., Chen X., Ma S., Xiao Z. (2021). Crosstalk between hepatic stellate cells and surrounding cells in hepatic fibrosis. Int. Immunopharmacol..

[22] Matsuda M., Seki E. (2020). Hepatic Stellate Cell-Macrophage Crosstalk in Liver Fibrosis and Carcinogenesis. Semin. Liver Dis..

[23] Friedman S.L. (2008). Hepatic stellate cells: Protean, multifunctional, and enigmatic cells of the liver. Physiol Rev..

[24] Park S.Y., Shin H.W., Lee K.B., Lee M.J., Jang J.J. (2010). Differential expression of matrix metalloproteinases and tissue inhibitors of metalloproteinases in thioacetamide-induced chronic liver injury. J. Korean Med. Sci..

[25] Roeb E. (2018). Matrix metalloproteinases and liver fibrosis (translational aspects). Matrix Biol..

[26] Geerts A. (2001). History, heterogeneity, developmental biology, and functions of quiescent hepatic stellate cells. Semin. Liver Dis..

[27] Cai X., Wang J., Wang J., Zhou Q., Yang B., He Q., Weng Q. (2020). Intercellular crosstalk of hepatic stellate cells in liver fibrosis: New insights into therapy. Pharmacol. Res..

[28] Lotowska J.M., Lebensztejn D.M. (2015). Immunoreactive hepatic stellate cells in biopsy material in children with hepatitis B: The first report in children. Pol. J. Pathol..

[29] Lotowska J.M., Sobaniec-Lotowska M.E., Lebensztejn D. (2018). M. Ultrastructural characteristics of the respective forms of hepatic stellate cells in chronic hepatitis B as an example of high fibroblastic cell plasticity. The first assessment in children. Adv. Med. Sci..

[30] Mehal W.Z., Azzaroli F., Crispe I.N. (2001). Immunology of the healthy liver: Old questions and new insights. Gastroenterology.

[31] Kmieć Z. (2001). Cooperation of Liver Cells in Health and Disease. Advances in Anatomy Embryology and Cell Biology Book Series.

[32] Puche J.E., Saiman Y., Friedman S.L. (2013). Hepatic stellate cells and liver fibrosis. Compr. Physiol..

[33] Elzamly S., Agina H.A., Elbalshy Abd El-Latif, Abuhashim M., Saad E., Abd Elmageed Z.Y. (2017). Integration of VEGF and α-SMA Expression Improves the Prediction Accuracy of Fibrosis in Chronic Hepatitis C Liver Biopsy. Appl. Immunohistochem. Mol. Morphol..

[34] Senoo H., Yoshikawa K., Morii M., Miura M., Imai K., Mezaki Y. (2010). Hepatic stellate cell (vitamin A-storing cell) and its relative--past, present and future. Cell. Biol. Int..

[35] Higashi T., Friedman S.L., Hoshida Y. (2017). Hepatic stellate cells as key target in liver fibrosis. Adv. Drug Deliver Rev..

[36] Friedman S.L., Sheppard D., Duffield J.S., Violette S. (2013). Therapy for fibrotic diseases: Nearing the starting line. Sci. Transl. Med..

[37] Campana L., Iredale J.P. (2017). Regression of liver fibrosis. Semin. Liver Dis..

[38] Schuppan D., Kim Y.O. (2013). Evolving therapies for liver fibrosis. J. Clin. Invest..

[39] Czaja A.J. (2014). Review article: The prevention and reversal of hepatic fibrosis in autoimmune hepatitis. Aliment. Pharmacol. Ther..

[40] Pellicoro A., Ramachandran P., Iredale J.P., Fallowfield J.A. (2014). Liver fibrosis and repair: Immune regulation of wound healing in a solid organ. Nat. Rev. Immunol..

[41] Zong Z., Liu J., Wang N., Yang C., Wang Q., Zhang W., Chen Y., Liu X., Deng H. (2021). Nicotinamide mononucleotide inhibits hepatic stellate cell activation to prevent liver fibrosis via promoting PGE(2) degradation. Free Radic. Biol. Med..

[42] Weiskirchen R., Tacke F. (2016). Liver Fibrosis: From Pathogenesis to Novel Therapies. Dig. Dis..

[43] Marrone G., Shah V.H., Gracia-Sancho J. (2016). Sinusoidal communication in liver fibrosis and regeneration. J. Hepatol..

[44] Lotowska J.M., Sobaniec-Lotowska M.E., Lebensztejn D.M., Daniluk U., Sobaniec P., Sendrowski K., Daniluk J., Reszec J., Debek W. (2017). Ultrastructural characteristics of rat hepatic oval cells and their intercellular contacts in the model of biliary fibrosis: New insights into experimental liver fibrogenesis. Gastroenterol. Res. Pract..

[45] Sobaniec-Lotowska M.E., Lotowska J.M., Lebensztejn D.M. (2007). Ultrastructure of oval cells in children with chronic hepatitis B, with special emphasis on the stage of liver fibrosis: The first pediatric study. World J. Gastroenterol..

[46] Sobaniec-Lotowska M.E., Lebensztejn D.M., Lotowska J.M., Kanczuga-Koda L., Sulkowski S. (2011). Ultrastructure of liver progenitor/oval cells in children with nonalcoholic steatohepatitis. Adv. Med. Sci..

[47] Lotowska J.M., Sobaniec-Lotowska M.E., Lebensztejn D.M. (2010). Electron microscopic alterations in intermediate hepatocyte-like cells in children with chronic hepatitis B. The first report in pediatric patients. Eur. J. Gastroenterol. Hepatol..

[48] Lotowska J.M., Sobaniec-Lotowska M.E., Lebensztejn D.M. (2013). The role of Kupffer cells in the morphogenesis of nonalcoholic steatohepatitis—ultrastructural findings. The first report in pediatric patients. Scand. J. Gastroenterol..

[49] Lotowska J.M., Sobaniec-Lotowska M.E., Bockowska S.B., Lebensztejn D.M. (2014). Pediatric non-alcoholic steatohepatitis: The first report on the ultrastructure of hepatocyte mitochondria. World J. Gastroenterol..

[50] Lotowska J.M., Sobaniec-Lotowska M.E., Sobaniec P., Lebensztejn D.M. (2018). Liver sinusoidal endothelial cells in morphogenesis of pediatric autoimmune hepatitis. Ultrastructural characteristics—A novel report. Pol. J. Pathol..

[51] Lotowska J.M., Sobaniec-Lotowska M.E., Sobaniec P. (2021). Ultrastructural Profile Combined with Immunohistochemistry of a Hepatic Progenitor Cell Line in Pediatric Autoimmune Hepatitis: New Insights into the Morphological Pattern of the Disease. Cells.

[52] Batts K.P., Ludwig J. (1995). Chronic hepatitis. An update on terminology and reporting. Am. J. Surg. Pathol..

[53] Ishak K. (1995). Histological grading and staging of chronic hepatitis. J. Hepatol..

[54] Gressner O.A., Lahme B., Demirci I., Gressner A.M., Weiskirchen R. (2007). Differential effects of TGF-beta on connective tissue growth factor (CTGF/CCN2) expression in hepatic stellate cells and hepatocytes. J. Hepatol..

[55] Beljaars L., Daliri S., Dijkhuizen C., Poelstra K., Gosens R. (2017). WNT-5A regulates TGF-β-related activities in liver fibrosis. Am. J. Physiol. Gastrointest. Liver Physiol..

[56] Tacke F., Zimmermann H.W. (2014). Macrophage heterogeneity in liver injury and fibrosis. J. Hepatol..

[57] Sasaki R., Devhare P.B., Steele R., Ray R., Ray R.B. (2017). Hepatitis C virus-induced CCL5 secretion from macrophages activates hepatic stellate cells. Hepatology.

[58] Tacke F. (2017). Targeting hepatic macrophages to treat liver diseases. J. Hepatol..

[59] Koyama Y., Brenner D.A. (2017). Liver inflammation and fibrosis. J. Clin. Invest..

[60] Pradere J.P., Kluwe J., De Minicis S., Jiao J.J., Gwak G.Y., Dapito D.H., Jang M.K., Guenther N.D., Mederacke I., Friedman R. (2013). Hepatic macrophages but not dendritic cells contribute to liver fibrosis by promoting the survival of activated hepatic stellate cells in mice. Hepatology.

[61] Oakley F., Meso M., Iredale J.P., Green K., Marek C.J., Zhou X., May M.J., Millward-Sadler H., Wright M.C., Mann D.A. (2005). Inhibition of inhibitor of kappaB kinases stimulates hepatic stellate cell apoptosis and accelerated recovery from rat liver fibrosis. Gastroenterology.

[62] Matsuda M., Tsurusaki S., Miyata N., Saijou E., Okochi H., Miyajima A., Tanaka M. (2018). Oncostatin M causes liver fibrosis by regulating cooperation between hepatic stellate cells and macrophages in mice. Hepatology.

[63] Guillot A., Tacke F. (2019). Liver macrophages: Old dogmas and new insights. Hepatol. Commun..

[64] Ahmad R., Ahmed S., Khan N.U., Hasnain A. (2009). Operculina turpethum attenuates N-nitrosodimethylamine induced toxic liver injury and clastogenicity in rats. Chem. Biol. Interact..

[65] Husain H., Latief U., Ahmad R. (2018). Pomegranate action in curbing the incidence of liver injury triggered by Diethylnitrosamine by declining oxidative stress via Nrf2 and NFκB regulation. Sci. Rep..

